# Global transcriptional response of oral squamous cell carcinoma cell lines to health-associated oral bacteria - an *in vitro* study

**DOI:** 10.1080/20002297.2022.2073866

**Published:** 2022-05-16

**Authors:** Divyashri Baraniya, Kumaraswamy Naidu Chitrala, Nezar Noor Al-Hebshi

**Affiliations:** a Oral Microbiome Research Laboratory, Maurice H. Kornberg School of Dentistry, Temple University, Philadelphia, USA; b Fels Cancer Institute for Personalized Medicine, Lewis Katz School of Medicine, Temple University, Philadelphia, Pennsylvania, USA; c Cancer Prevention and Control Program, Fox Chase Cancer Center, Temple University Health System, Philadelphia, USA

**Keywords:** Mouth neoplasms, cell line, bacteria, microarray analysis, transcriptome

## Abstract

**Background:**

We have recently demonstrated that health-associated oral bacteria *Streptococcus mitis*, *Neisseria flavescens*, and *Haemophilus parainfluenzae* induce cytotoxicity in oral squamous cell carcinoma (OSCC) cell lines and downregulate CD36, a cancer-assocaited gene.

**Aim:**

To explore the effect of these three species on global transcriptome of OSCC cell lines.

**Methods:**

Gene expression of cell lines CAL27, SCC4 and SCC25 cocultured with the test species was assessed with Clariom-S Human microarray. *Porphyromonas gingivalis* was included as a pathogenic control. Data were analyzed using Ingenuity Pathway Analysis.

**Results:**

The results differed by species and cell line. Overall, the transcriptional changes by *S. mitis* were predominantly anti-cancer including inhibition of HOTAIR regulatory pathway, JAK/Stat signaling, cyclins/cyclin-dependent kinases, and endothelin1 signaling. *H. parainfluenzae* and *N. flavescens* resulted in a mix of pro- and anti-cancer responses including activation of acute phase response, pro-inflammatory interleukins signaling, TREM-1 signaling, and tumor microenvironment pathway; but downregulation of cell cycle by inhibition of cyclins and cyclin-dependent kinases. *P. gingivalis* had a predominantly pro-cancer effect limited to SCC4, including upregulation of inflammatory pathways, phospholipases and PI3K signaling.

**Conclusion:**

These findings provide a new insight into the role of commensal oral bacteria in OSCC. Animal studies are required to further explore them.

## Introduction

Oral squamous cell carcinoma (OSCC) is the most common type of malignancy affecting the mouth. Tobacco and alcohol use are the major risk factors for OSCC, but a subset is caused by Human Papilloma Virus; other known risk factors include chronic inflammation, immunosuppression, genetic predisposition and diet [1]. Recently, there has been growing interest in the role of the oral microbiome in OSCC [2,3]. Results from a plethora of epidemiological studies demonstrate that the microbiome associated with OSCC is distinct from that associated with health [2,3]. While there is no consistent microbial signature, a number of bacterial taxa, particularly *Fusobacterium* spp., have been repeatedly found to be overabundant in OSCC [4–7]. *In vitro* and animal studies demonstrate that oral pathogens such as *Fusobacterium nucleatum* and *Porphyromonas gingivalis* can contribute to oral carcinogenesis by various mechanisms including increasing cellular proliferation, inhibiting apoptosis, inducing chronic inflammation and promoting cellular invasion [2].

In exploring the role of the microbiome in oral cancer, researchers seem to have almost exclusively focused on studying the bacterial species enriched in the OSCC, i.e. the pathobionts, while largely ignoring the health-associated species, when epidemiological studies also show several species to be consistently enriched in the control samples [2,3]. Some of these species such as *Streptococccus* spp, *Hemophilus parainfluenzae, Veilonella parvula* have even been shown to negatively correlate with staging of OSCC [8]. Commensal bacteria have a role not only in sustaining healthy immune functions but also provide defense against colonization of pathogenic bacteria and help enhancing selective immunity against tumor tissues [9,10]. Furthermore, the microbiome has the capacity to modulate the effect of cancer treatments and can even improve treatment outcomes [11]. In the context of colon cancer for example, evidence suggest that certain gut bacteria such as *Clostridium, Bifidobacterium, Listeria, Escherichia coli*, and *Salmonella* species have anti-cancer properties, which may provide a distinctive therapeutic option with less side effects [10].

In the context of oral cancer, a recent study by Ohshima et al. found *Streptococcus gordonii* (a health-associated oral bacteria) to antagonize the expression of pro-cancer molecule/ZEB2 induced by *Porphyromonas gingivalis* in oral epithelial cells [12], suggesting it may have a protective role against oral cancer. In another study [13], we have recently screened six oral health-associated bacteria, namely *Streptococcus mitis, Hemophilus parainfluenzae, Neisseria flavescens, Rothia mucilaginosa, Lautropia mirabilis* and *Veilonella parvula* for their effect on proliferation and expression of marker genes in OSCC cell lines CAL27, SCC4 and SCC25. These taxa have consistently been found in OSCC microbiome studies to be enriched in samples from healthy subjects compared to those from oral cancer patients [6,7,14–19]. Out of these six species, *S. mitis, H. parainfluenzae* and *N. flavescens* demonstrated dose-dependent inhibition of proliferation, which was found to be mediated by H_2_O_2_ for *S. mitis* and by intracellular infection for *H. parainfluenzae* and *N. flavescens*. All three species also downregulated *CD36*, a gene involved in tumor metastasis and growth [20]. On the other hand, they upregulated expression of *IL6* and *TNF*, which are known to contribute to carcinogenesis by mediating inflammation [21,22]. The objective of this study was, therefore, to have a more comprehensive insight into the interaction between these three species and OSCC cell lines by performing global transcriptome analysis. *P. gingivalis* was included as a pathogenic control.

## Methods

### OSCC cell lines and bacterial strains

OSCC cell lines CAL27, SCC4 and SCC25 were acquired from the American Tissue Culture Collection (ATCC), certified as mycoplasma-free and STR-authenticated. The cell lines were grown in 5% CO_2_ at 37°C in Dulbecco’s modified Eagle’s medium (DMEM) supplemented with 10% fetal bovine serum (FBS) and 2.5 mM L-glutamine (CAL27) or a 1:1 mixture of DMEM and Ham’s F12 medium containing 2.5 mM L-glutamine, 400 ng/ml hydrocortisone and 10% FBS (SCC4 and SCC25).

Bacterial strains *S. mitis ATCC 49456, N. flavescens ATCC 13120* and *P. gingivalis ATCC 33277* were obtained from ATCC whereas *H. parainfluenzae NCTC 10665* was obtained from Public Health England. *P. gingivalis* was included as a pathogenic control given existing evidence on its carcinogencity [2,3]. Brain Heart Infusion (BHI) supplemented with 0.5% hemin, 0.1% Vitamin K and 1% Isovitalex was used as a culture medium for all bacteria. The health-associated strains were grown at 37°C in 5% CO_2_; *P. gingivalis* was grown at 37°C in anaerobic conditions (10% hydrogen, 10% CO_2_, and 80% nitrogen).

### Co-culturing bacteria with OSCC cell lines

This was done as described previously [13]. Briefly, OSCC cells were seeded at 25,000–35,000 cells/well, depending on the cell line, in 48 well plates (TPP, Switzerland). The cells were allowed to attach for 24 h before bacteria grown to mid-log stage were added at a multiplicity of infection (MOI) of 100 – this concentration was selected based on results from our previous study in which MOI of 100 showed the highest upregulation of selected genes [13]. The co-cultures were then incubated for additional 24 hours. For each strain, a sub-minimum inhibitory concentration (sub-MIC) of streptomycin/penicillin was used to control bacterial overgrowth, as previously demonstrated [13]. All the co-cultures were performed in technical triplicates. Cells treated with culture medium devoid of bacteria were used as negative control.

### RNA extraction and microarray analysis

At the end of the 24 h co-culture incubation period, the culture medium was removed, the cells were washed with PBS, and RNA was isolated using the PureLink RNA extraction kit (Invitrogen, USA) following manufacturer’s instructions. SUPERase·In^TM^ RNase inhibitor (Invitrogen, USA) was added to the purified RNA at a concentration of 1 U/μl of RNA and contaminating DNA was digested using the Turbo DNA-free^TM^ kit (Invitrogen, USA). Yield and quality were assessed using a Nanodrop (ThermoFisher Scientific, USA). RNA was stored at −80°C untill further processing.

Microarray assays were carried out at Thermo Fisher Scientific (USA) using Affymetrix Clariom S Human chip according to the manufacturer’s protocol. The raw data was deposited at NCBI’s Gene Expression Omnibus (GSE183911). Array quality control, data normalization and identification of differentially expressed genes (DEGs) were performed using Applied Biosystems^TM^ Transcriptome Analysis Console (TAC) with an integrated *LIMMA* package [23] for statistical analyses. DEGs were defined as those with fold change greater than 1.5 in either direction and false discovery rates (FDR) ≤0.1. Venn analysis to find common genes between the different cell lines with same treatment was performed using Venn Diagrams tool by Van De Peer Lab (http://bioinformatics.psb.ugent.be/webtools/Venn/). Gene enrichment analysis, including identification of canonical pathways, causal networks, upstream regulators and biological functions was performed using Ingenuity Pathway AAnalysis (Qiagen Inc., https://www.qiagenbioinformatics.com/products/ingenuitypathway-analysis). Significantly altered pathways and biological functions were defined as those with a p-value ≤0.05 and activation status z-score ≥2 or ≤-2. For pathways, the changes induced by the test strains were predicted to be pro-cancer, anti-cancer or ambiguous based on the known association of the particular pathway with cancer and the direction of the respective z-score. For example, an upregulation (positive z-score) of a pathway known to be activated in cancer (e.g. tumor microenvironment pathway) was predicted as a pro-cancer effect, while down-regulation (negative z-score) of a pathway known to be inhibited in cancer (e.g. apoptosis) was predicted as an anti-cancer effect. For pathways for which evidence about association with cancer is conflicting, then the change was predicted as ambiguous.

## Results

### Differentially expressed genes (DEGs)

**Figure 1.**
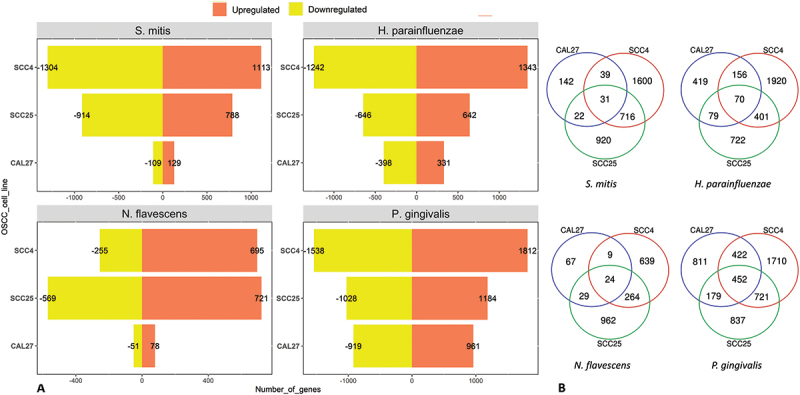
The number of differentially expressed genes by bacterium and cell line. Microarray data obtained from co-cultures of OSCC cell lines with each of the test bacterial species were analyzed with Transcriptome Analysis Console to identify differentially expressed genes (DEGs) defined as those with 1.5-fold change in either direction and false discovery rates (FDR) ≤0.1. **A)** The number of upregulated and downregulated DEGs in each co-culture. B) A Venn diagram showing the number of exclusive and shared DEGs between the three cell lines for each bacterial treatment.

**Figure 2.**
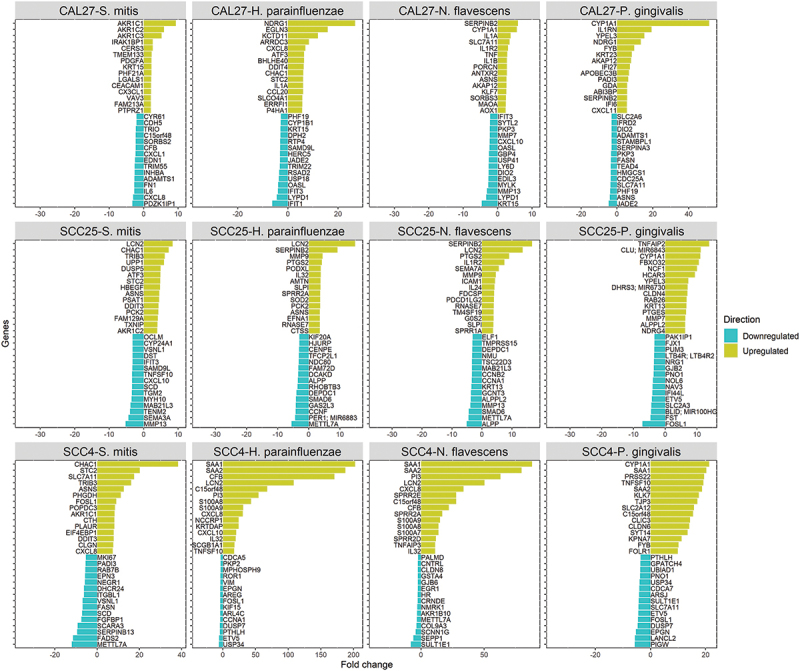
Top differentially expressed genes (DEGs). The 15 top upregulated and 15 top downregulated genes in each co-culture. DEGs were identified as described in the text and legend of Figure 1.

The number of DEGs by bacterium and cell line are presented in Figure 1(a). Lists of these genes and along with fold change and other details are provided in **Supplementary File 1**. *P. gingivalis* resulted in the highest number of DEGs in all cell lines, while CAL27 showed the lowest number of DEGs for all the bacterial strains. For CAL27 treated with *S. mitis*, only 20 DEGs were initially identified at the FDR cutoff value of 0.1, so for this co-culture the threshold was relaxed to a nominal p-value of 0.01 to obtain more DEGs for downstream analysis, which resulted in a total of 236 DEGs. For each bacterium, the DEGs varied by cell line, with the highest overlap observed between SCC4 and SCC25 as shown in Figure 1(b). The list of exclusive and shared DEGs among the cell lines for each bacterium is provided in **Supplementary File 2**. Figure 2 shows the 15 top upregulated and 15 top downregulated genes by the test bacterial strains in each of the cell lines.

### *Transcriptional changes by* S. mitis *are predominantly anti-cancer*

**Figure 3.**
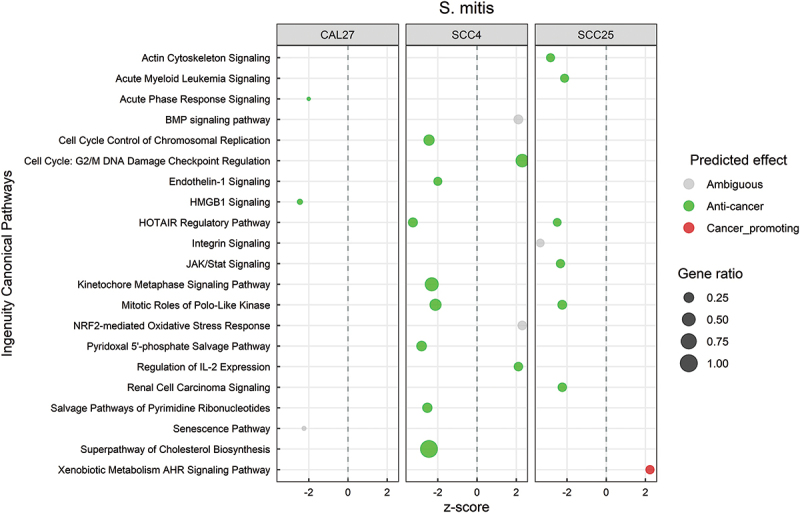
Canonical pathways significantly altered by S*treptococcus mitis*. Microarray data obtained from co-cultures of OSCC cell lines with *S. mitis* were analyzed with Transcriptome Analysis Console to identify differentially expressed genes (DEGs), which were in turn processed with Ingenuity Pathway Analysis to identify significantly upregulated and downregulated pathways (P ≤ 0.05; z-scores ≤ −2 or ≥ 2). The effect (red/green) was predicted based on the direction (z score) and known association of the particular pathway with cancer (see text for more details). Gene ratio indicates number of genes from the list that maps to a particular pathway divided by the total number of genes that map to the same pathway.

Canonical pathways significantly regulated by *S. mitis* (P ≤ 0.05, with z-scores ≤ −2 or ≥ 2) are shown in Figure 3, and the DEGs corresponding to each pathway are listed in **Supplementary File 3**. There was little overlap in the responses between the three cell lines, but the changes were predominantly anti-cancer, regardless. These included inhibition of HOTAIR regulatory pathway; control of cell cycle by downregulation of cyclins, cyclin-dependent kinases, polo-like kinases and metaphase signaling; downregulation of inflammatory pathways including JAK/Stat, endothelin1, HMGB1 and acute phase response; and downregulation of cholesterol biosynthesis. At the functional level, *S. mitis* inhibited cancer-associated functions like cell proliferation, angiogenesis, invasion, metastasis, and migration/movement of cells (Supplementary Figure S1), the DEGs contributing to these functions are provided in **Supplementary File 4**.

### H. parainfluenzae *and N*. flavescens *induce a mix of anti-cancer and pro-cancer responses*

**Figure 4.**
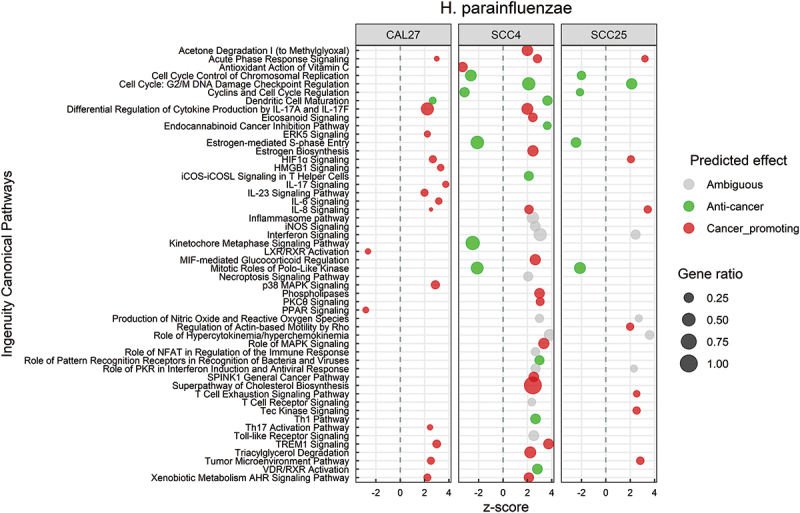
Canonical pathways significantly altered by *Haemophilus parainfluenzae*. Microarray data obtained from co-cultures of OSCC cell lines with *H. parainfluezae* were analyzed with Transcriptome Analysis Console to identify differentially expressed genes (DEGs), which were in turn processed with Ingenuity Pathway Analysis to identify significantly upregulated and downregulated pathways (P ≤ 0.05; z-scores ≤ −2 or ≥ 2). The effect (red/green) was predicted based on the direction (z score) and known association of the particular pathway with cancer (see text for more details). Gene ratio indicates number of genes from the list that maps to a particular pathway divided by the total number of genes that map to the same pathway.

Canonical pathways that were significantly affected by *H. parainfluenzae* in the three cell lines are illustrated in Figure 4. The DEGs involved in each of these pathway are listed in **Supplementary File 5**. The altered pathways differed by cell line, but there was a considerable overlap between SCC4 and SCC25. Both pro-cancer and anti-cancer effects were observed. The pro-cancer changes involved primarily upregulation of pathways involved in inflammation, e.g. IL-6, IL-8, IL-17, HMGB1, TREM-1 and tumor microenvironment signaling, while the anti-cancer effects were predominantly related to control of cell cycle through downregulation of cyclins, cyclin-dependent kinases, polo-like kinase and metaphase signaling pathway. The biological functions significantly altered by *H. parainfluenzae* are presented in Supplementary Figure S2 and the corresponding DEGs are listed in **Supplementary File 6**. Again, the altered functions varied by cell line and were mixed in nature. For example, cell proliferation of carcinoma cell lines was inhibited, while cell movement, invasion and angiogenesis were activated.

**Figure 5.**
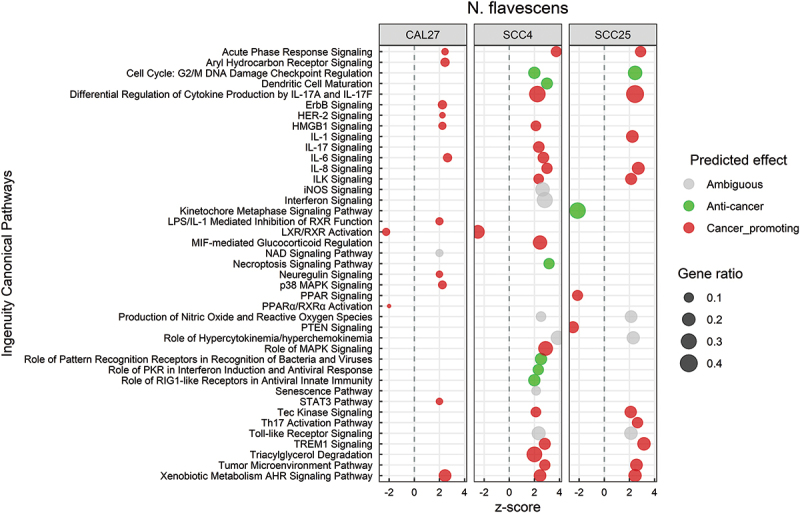
Canonical pathways significantly altered by *Neisseria flavescens*. Microarray data obtained from co-cultures of OSCC cell lines with *N. flavescens* were analyzed with Transcriptome Analysis Console to identify differentially expressed genes (DEGs), which were in turn processed with Ingenuity Pathway Analysis to identify significantly upregulated and downregulated pathways (P ≤ 0.05; z-scores ≤ −2 or ≥ 2). The effect (red/green) was predicted based on the direction (z score) and known association of the particular pathway with cancer (see text for more details). Gene ratio indicates number of genes from the list that maps to a particular pathway divided by the total number of genes that map to the same pathway.

Response to *N. flavescens* was dominated by activation of inflammatory pathways including IL-1, IL-6, IL-8, IL-17, STAT3, HMGB1, TREM-1 and tumor microenvironment signaling (Figure 5; see **Supplementary File 7** for the corresponding DEGs). Other pro-cancer changes included activation of ErbB/Her-2 signaling, and inhibition of LXR, RXR and PPAR pathways. On the other hand, *N. flavescens* also induced anti-cancer transcriptional changes including inhibition of cyclins/cyclin-dependent kinases and metaphase signaling, and activation of necroptosis. At the biological function level (Supplementary Figure S3), however, the effects *N. flavescens* were predominantly on the pro-cancer side including increase of cellular migration and invasion, angiogenesis, vasculogenesis, and lipid metabolism. The DEGs involved in these biological processes are listed in **Supplementary File 8**).

### P. gingivalis *elicit a predominantly pro-cancer response in SCC4 but not in SCC25 and CAL27*

**Figure 6.**
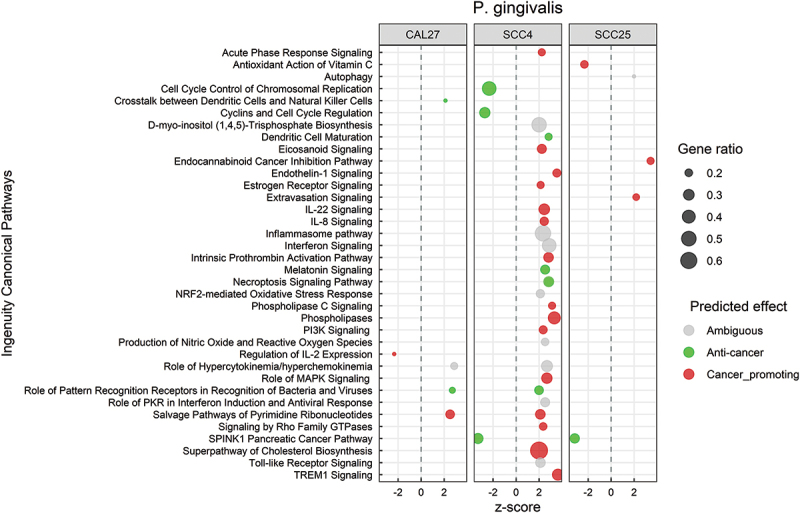
Canonical pathways significantly altered by *Porphyromonas gingivalis*. Microarray data obtained from co-cultures of OSCC cell lines with *P. gingivalis* were analyzed with Transcriptome Analysis Console to identify differentially expressed genes (DEGs), which were in turn processed with Ingenuity Pathway Analysis to identify significantly upregulated and downregulated pathways (P ≤ 0.05; z-scores ≤ −2 or ≥ 2). The effect (red/green) was predicted based on the direction (z score) and known association of the particular pathway with cancer (see text for more details). Gene ratio indicates number of genes from the list that maps to a particular pathway divided by the total number of genes that map to the same pathway.

Figure 6 shows the significantly altered canonical pathways by *P. gingivalis*; the corresponding DEGs are included in **Supplementary File 9**. Transcriptional changes at the pathway level were primarily observed in SCC4 and were mostly pro-cancer in nature. This included upregulation of inflammation-related pathways such as acute phase response, IL-22, IL-8, TREM1, and endothelin1 signaling, and activation of phospholipases, PI3K and cholesterol biosynthesis pathways. On the other hand, cyclins and cyclin-dependent kinases were downregulated. At the biological functions level, the changes were pro-cancer for SCC4, consistent with the pathway-level results (Supplementary Figure S4). However, anti-cancer responses were identified in CAL27 and SCC25 including increased apoptosis and necrosis and decreased cellular proliferation and invasion. DEGs corresponding to these biological functions are provided in **Supplementary File 10**.

### Results validation and summary

For validation of the microarray results, we performed qPCR assays for three genes, namely LCN2, CXCL8 and MMP13 that were found to be significantly upregulated or downregulated by microarray analysis. The qPCR assays were done using predesigned Taqman primer/probe sets (ThermoFisher Scientific, USA) in a one-step reaction as described elsewhere [13]. The results were consistent with those obtained from the microarray analysis as shown in Supplementary Table S1, although the latter tended to over-estimate the magnitude of fold-change.

**Figure 7.**
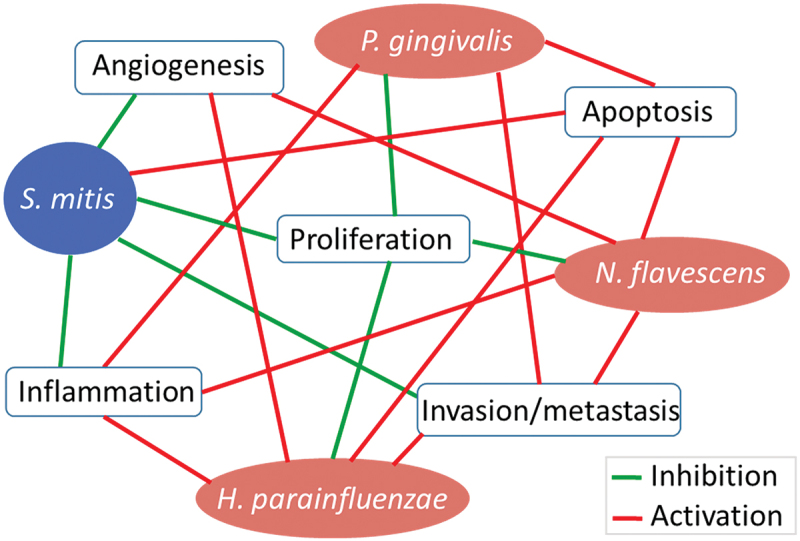
High-level summary. A summary of major transcriptional changes induced by the test species in the OSCC cell lines, incorporating results from IPA and GSEA analyses.

To validate and supplement the results obtained with IPA, we performed additional analysis of the microarray data with Gene Set Enrichment Analysis (GSEA) [24]. The results from the latter are presented in Supplementary Figures S5–8. While differences in the naming of the pathways between the two platforms made direct comparison difficult, the GSEA and IPA results were largely consistent at the high level (e.g. proliferation or inflammation). For example, GSEA showed that *S. mitis* down-regulates epithelilial-to-mesenchymal transition (EMT) while the other three species upregulated it, which is consistent with IPA’s results at the biological function level (migration, invasion and metastasis). GSEA also provided additional information, for example upregulation of apoptosis by all test species. A high-level summary of the results from IPA and GSEA is provided in Figure 7.

## Discussion

OSCC tissues are exposed to a complex microbial community, where different species may play apposing roles. For a better understanding of the individual roles played by oral microorganisms in OSCC, experiments involving mono-species, although an oversimplification, are inevitable. There are numerous such studies in the literature, but the focus has been primarily on pathogenic species. To address this gap, we have been working on a project, including the current study, to explore the potential protective role of health-associated oral bacteria in OSCC *in vitro* using a panel of cell lines. 2D cell culture models where a confluent monolayer of cells is treated with bacteria provides simplicity and an advantage of studying direct cellular responses to specific interactions with selected bacteria in high throughput format [25]. However, these systems come with limitations primarily that they are devoid of tumor microenvironment, host immune cells, and stromal components, so how the test species interact with and modulate the immune response and cell-to-cell signaling cannot be assessed; growing in plastic wells is also associated with change in cellular polarity and gene expression [26]. Additionally, in co-cultures, bacteria can easily overgrow and destroy the cancer cells; while we accounted for that by adding sub-MIC conentrations of antibiotics to the media to control bacterial growth; that may have modified the effect of the test species on the cell line. Therefore, the results from this study should be interpreted causiously and validated in more robust models.

Deregulation of cell cycle controls is fundamental to cancer cells and thus many recent therapies target cell cycle proteins [27]. The test bacteria affected cell cycle events in cell lines SCC4 and SCC25. *S. mitis, H. parainfluenzae* and *N. flavescens* activated G2/M DNA damage checkpoint regulation and downregulated chromosomal replication, cyclins and cell cycle regulation, estrogen-mediated S-phase entry, kinetochore metaphase signaling pathway and mitotic role of polo-like kinase. G2/M DNA damage checkpoint regulation prevents a cell to undergo mitosis when DNA is damaged and cancer cells often have an aberrant G2/M DNA damage checkpoint [28]. Cell cycle-related pathways in SCC4 and SCC25 cell lines were associated with downregulation of genes coding for various cyclins (CCNs), cyclin-dependent kinases (CDKs), origin recognition complex (ORC), *POLA1* and *TOP2A* genes. These genes play key roles in regulating cell cycle and deregulation of one or more of these are often associated with an increased proliferation of cells, and inhibition of these genes is widely considered for anti-tumor therapies [29–32]. Moreover, there was an upregulation of *CDKN1* gene, which encodes a tumor suppressor protein and is also a target in therapies for many cancers [33–35]. Some of cell cycle pathways were also associated with a downregulation of polo-like kinases PLK1 and PLK2; the roles of these in cancer is quite controversial where findings suggest both oncogenic and tumor suppressor roles in various cancers [36]. Overall, in the context of cell cycle regulation, the transcriptional changes were on the anti-cancer side.

Interleukins (IL) play a significant role in cancer. The specific cytokines present in the tumor microenvironment (TMN) determine if a pro-tumor inflammatory or anti-tumor immune response dominates [37]. In this study, *S. mitis* activated signaling of IL-2, an interleukin known to enhance anti-tumor immunity [38]. *S. mitis* also down-regulated the inflammatory pathways JAK/Stat, HMGB1 and acute phase response, which are known to be involved in tumor progression [39–41]. Furthermore, it downregulated matrix metalloproteases (MMPs) 1, 7 and 13 (except in CAL27), which are known to contribute to tumor invation and metastasis [42]. Furthermore, *S. mitis* inhibited the oncogenic HOTAIR [43] and cholesterol biosynthesis pathways [44]. Overall, these effects are consistent with anti-cancer properties.

On the other hand, *H. parainfluenzae* and *N. flavescens* activated signaling pathways for IL-1, IL-6, IL-17, IL-8 and IL-23, all of which are known to be pro-inflammatory [37]. They also upregulated other inflammatory pathways including HMGB1 and TREM-1, while their effect on MMPs differed by cell line. However, these finding should be interpreted with cautions since interleukins can play dual roles in cancer. For example, while IL-6 contributes to carcinogenesis by stimulating proliferation and sustaining chronic inflammation, it has been also found to mobilize anti-tumor T cell immune responses [45]. Similarly, while IL-17 is known to bolster tumor development, it has been observed to have antitumor properties in some cancer types include those of the head and neck [46]. In fact, *H. parainfluenzae* also activated T helper 1 pathway, which plays an important role in anti-cancer immunity [46]. The pathogenic *P. gingivalis* activated the inflammatory pathways IL-22, IL-8, TREM1, and endothelin1 signaling, downregulated IL-2 signaling, and consistent with previous studies [3], upregulated MMPs.

Nuclear receptors also play key roles in inflammation and cancer [47,48]. In this study, *H. parainfluenzae* and *N. flavescens* downregulated LXR/RXR, PPARα/RXRα and PPAR signaling, which are known to have tumor suppressive effect [47]. On the other hand, *H. parainfluenza* also upregauted VDR/RXR signaling in SCC4, which has potential to control cancer cell growth by driving anti-proliferative pathways and by enhancing adhesion [49]. Furthermore, despite their potential anti-cancer roles, many of the NRs are also overexpressed in specific cancers [47,50]. *S. mitis* activated NRF2, which mediates antioxidant responses and thus plays a role in cancer prevention; however, there are also studies that implicate it in cancer progression [51]. Therefore, there is significant ambiguity about the nature of many of the observed transcriptional changes, i.e. it is not straightforward to predict whether they represent anti- or pro-cancer effects. There were other examples of such ambiguous changes. *H. parainfluenzae, N. flavescens* and *P. gingivalis*, upregulated nitric oxide and reactive oxygen species, which have a dual role in cancer depending on their concentration in the TME [52]. Similarily, Acetone degradation I (to Methyglyoxal) pathway, which was activated by *H. parainfluenzae* and *N. flavescens*, has recently been suggested to play a dual role in cancer depending on the rate of production [53].

*P. gingivalis* has shown to possess carinogenic properties *in vitro* and in animal models [2]. Consistent with that, this study found *P. gingivalis* to activate pro-inflmmatory pathways and MMPs as discussed above. It also activated known oncogenic pathways including phospholipases [54], PI3K [55] and cholesterol biosynthesis pathways [44]. Some of these effects have been demonstrated in previous studies [56,57], consistent with pro-cancer properties. However, in this study, the effects were limited to SCC4, suggesting *P. gingivalis* may have exerted its effect through interaction with receptors expressed by SCC4 but not the other two cell lines. This also suggests *P. gingivalis* may play a role in progression of only a subset of oral cancers. It is worth mentioning that that some of the transcriptional changes induced by *P. gingivalis* were also observed with *H. parainfluenzae* or *N. flavescens*, indicating these changes are not specific to *P. gingivalis*. This raises concerns about the specificity of carcinogenic properties previously reported for *P. gingivalis* and emphasizes the importance of including Gram-negative health-associated species as controls.

In conclusion, *S. mitis* was found here to induce predominantly anti-cancer transcriptional changes, while *H. parainfluenzae* and *N. flavascens* elicited both anti- and pro-cancer responses. However, given its preferential cytotoxicity against OSCC cancer cell lines that we demonstrated in a previous study, *H. parainfluenzae* remains a candidate as anti-cancer species besides *S. mitis* that is worth exploring in future studies. We do realize that *in vitro* cell culture work has inherent limitations including lack of tumor microenvironment, host immune/inflammatory response, and vasculature. Therefore, testing in an animal model of OSCC is crucial to better understand the interaction between these health-associated oral bacterial species and host cells in the context of oral carcinogenesis

## Supplementary Material

Supplemental Material
