# Supplementary material for: Global transcriptional response of oral squamous cell carcinoma cell lines to health-associated oral bacteria - an in vitro study
Source: J Oral Microbiol. 2022 May 16;14(1):2073866. doi: 10.1080/20002297.2022.2073866 (PMC9116255; doi:10.1080/20002297.2022.2073866)
Supplement: Supplemental Material [file ZJOM_A_2073866_SM9106.docx]

**Supplementary tables and figures**

**Supplementary Table 1.** Validation of gene expression results with qPCR.

| Gene Symbol | Cell line | Bacteria | RT qPCR Fold Change | RNA microarray Fold Change |
| --- | --- | --- | --- | --- |
| LCN2 | SCC4 | *H. parainfluenzae* | 41.11 | 108.35 |
| CXCL8 | SCC4 | *H. parainfluenzae* | 17.75 | 30.42 |
| MMP13 | SCC25 | *H. parainfluenzae* | -2.5 | -2.75 |
| LCN2 | SCC4 | *N. flavescens* | 18.45 | 50.37 |
| CXCL8 | SCC4 | *N. flavescens* | 17.99 | 33.57 |
| MMP13 | SCC25 | *N. flavescens* | -2.82 | -3.86 |
| LCN2 | SCC4 | *S. mitis* | 3.02 | 3.44 |
| CXCL8 | SCC4 | *S. mitis* | 5.84 | 7.35 |
| MMP13 | SCC25 | *S. mitis* | -3.72 | -5.28 |
| LCN2 | SCC4 | *P. gingivalis* | 6.58 | 8.13 |
| CXCL8 | SCC4 | *P. gingivalis* | 3.71 | 2.99 |
| MMP13 | SCC25 | *P. gingivalis* | 4.01 | 4.99 |

**
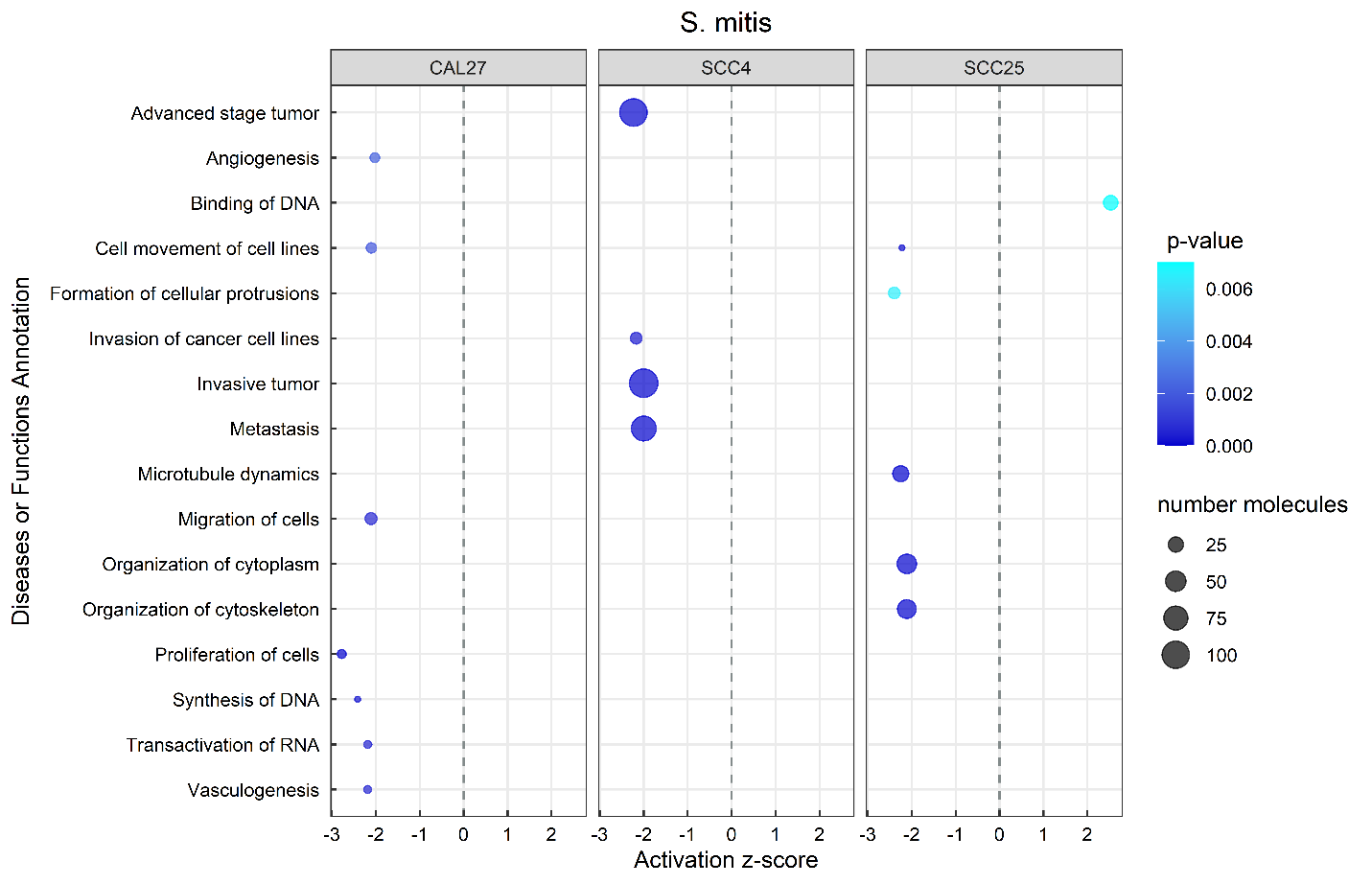
**

**Supplementary Figure 1.**  **Biological functions significantly altered by S*treptococcus mitis***. Microarray data obtained from co-cultures of OSCC cell lines with S. mitis were analyzed with Transcriptome Analysis Console to identify differentially expressed genes (DEGs), which were in turn processed with Ingenuity Pathway Analysis to identify significantly upregulated and downregulated biological functions (P ≤ 0.05; z-scores ≤ -2 or ≥ 2). Number of molecules are the number of genes associated with that Disease or Biological function.

**
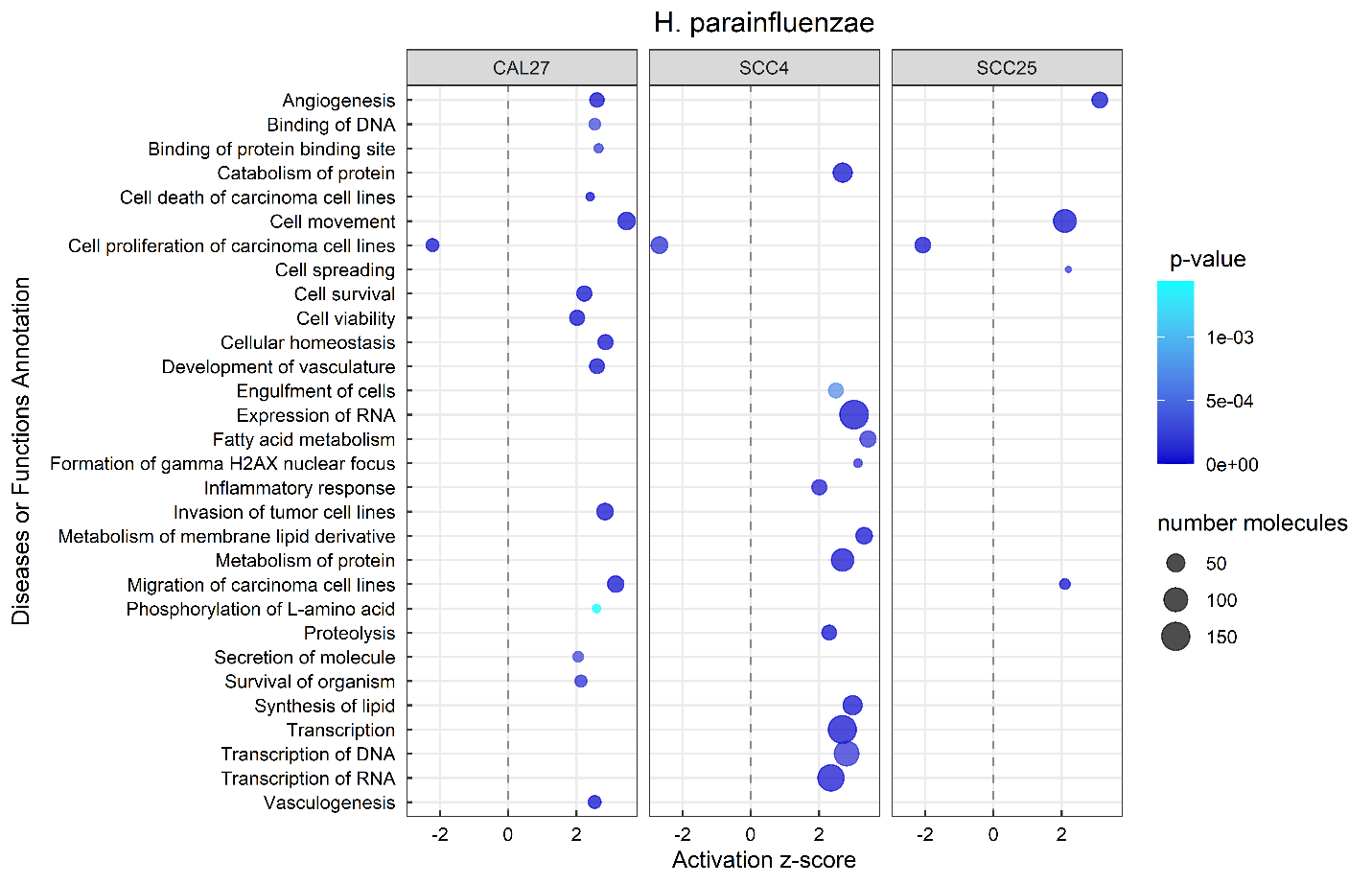
**

**Supplementary Figure 2.**  **Biological functions significantly altered by *Haemophilus parainfluenzae***. Microarray data obtained from co-cultures of OSCC cell lines with H*. parainfluenzae* were analyzed with Transcriptome Analysis Console to identify differentially expressed genes (DEGs), which were in turn processed with Ingenuity Pathway Analysis to identify significantly upregulated and downregulated biological functions (P ≤ 0.05; z-scores ≤ -2 or ≥ 2). Number of molecules are the number of genes associated with that Disease or Biological function.


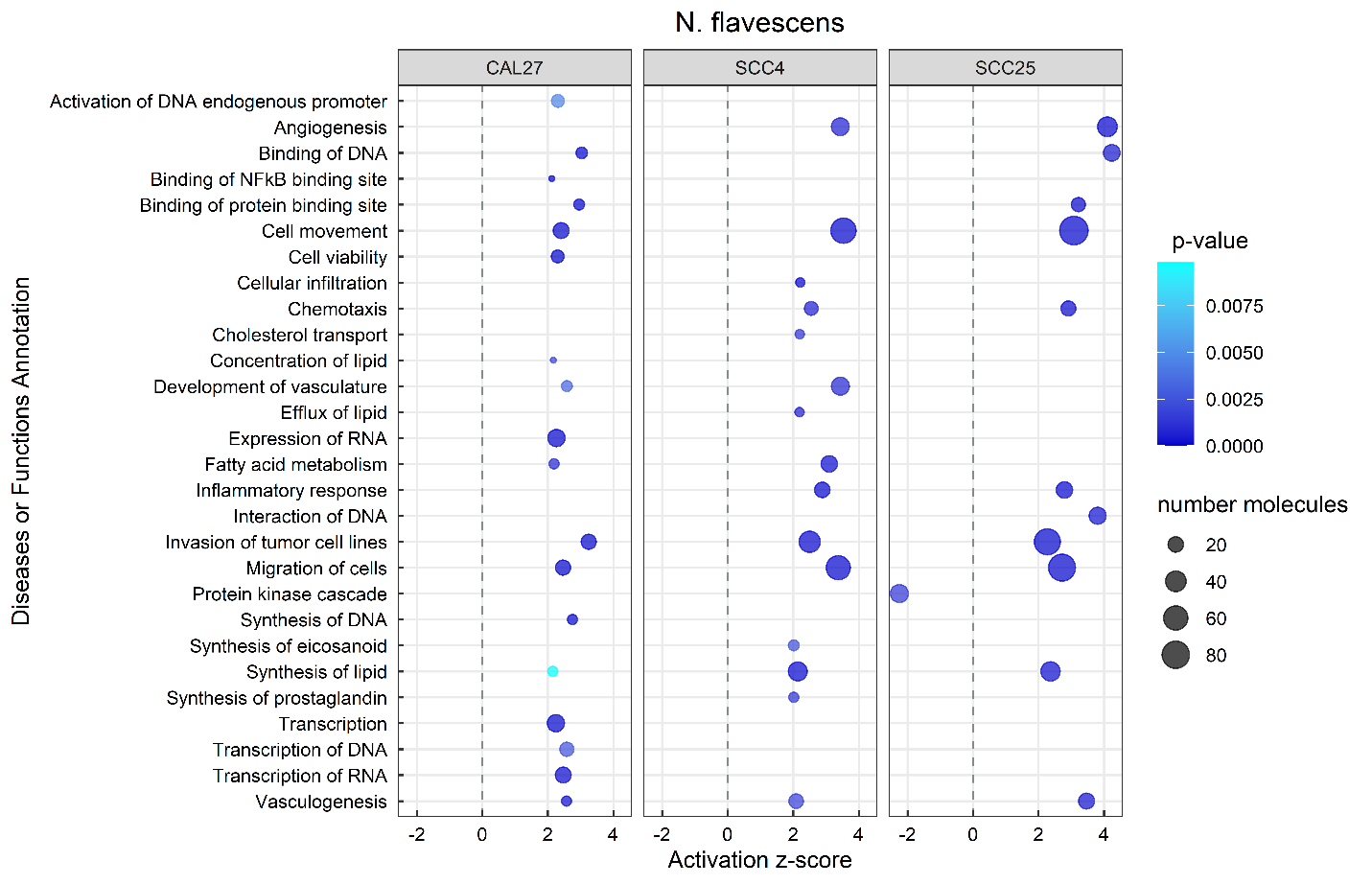


**Supplementary Figure 3.** **Biological functions significantly altered by *Neisseria flavescens***. Microarray data obtained from co-cultures of OSCC cell lines with *N. flavescens* were analyzed with Transcriptome Analysis Console to identify differentially expressed genes (DEGs), which were in turn processed with Ingenuity Pathway Analysis to identify significantly upregulated and downregulated biological functions (P ≤ 0.05; z-scores ≤ -2 or ≥ 2). Number of molecules are the number of genes associated with that Disease or Biological function.

**
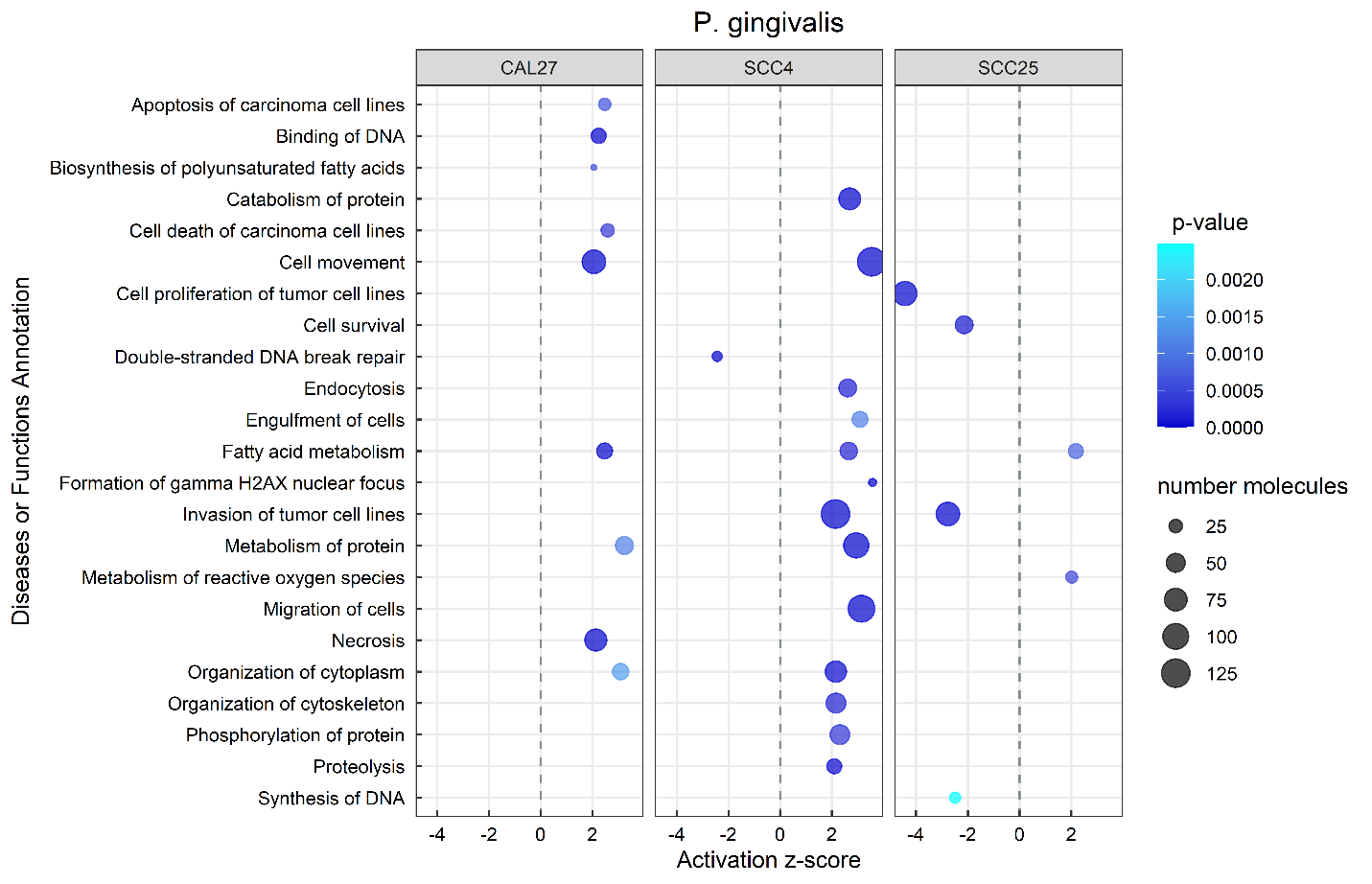
**

**Supplementary Figure 4.**  **Biological functions significantly altered by *Porphyromonas gingivalis***. Microarray data obtained from co-cultures of OSCC cell lines with *P. gingivalis* were analyzed with Transcriptome Analysis Console to identify differentially expressed genes (DEGs), which were in turn processed with Ingenuity Pathway Analysis to identify significantly upregulated and downregulated biological functions (P ≤ 0.05; z-scores ≤ -2 or ≥ 2). Number of molecules are the number of genes associated with that Disease or Biological function.


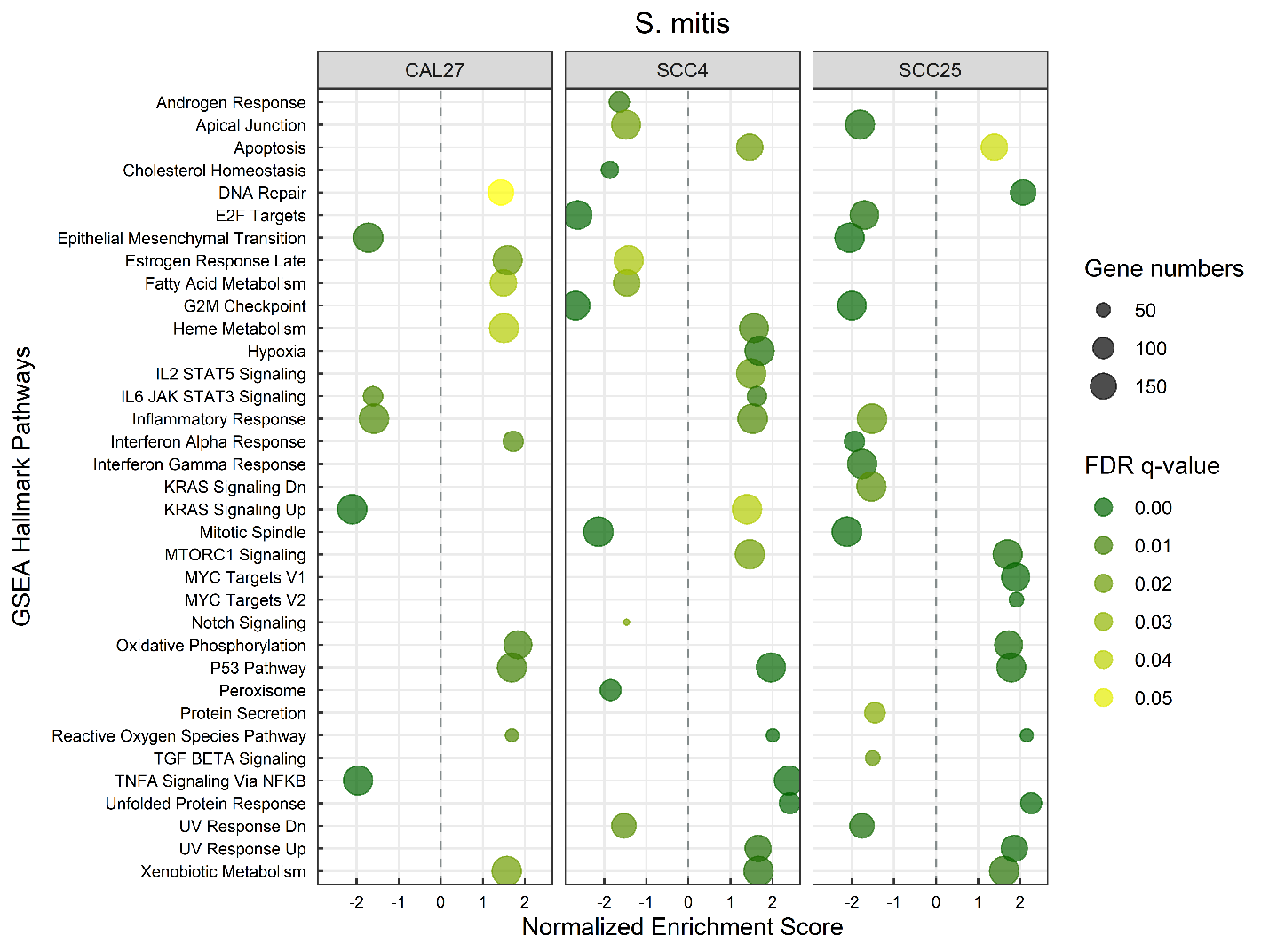


**Supplementary Figure 5.**  **GSEA Hallmark Pathways significantly altered by S*treptococcus mitis***. Microarray data obtained from co-cultures of OSCC cell lines with S. mitis were analyzed with Transcriptome Analysis Console to identify differentially expressed genes (DEGs), which were in turn processed with Gene Set Enrichment Analysis (GSEA) to identify significantly upregulated and downregulated pathways (FDR ≤ 0.05).


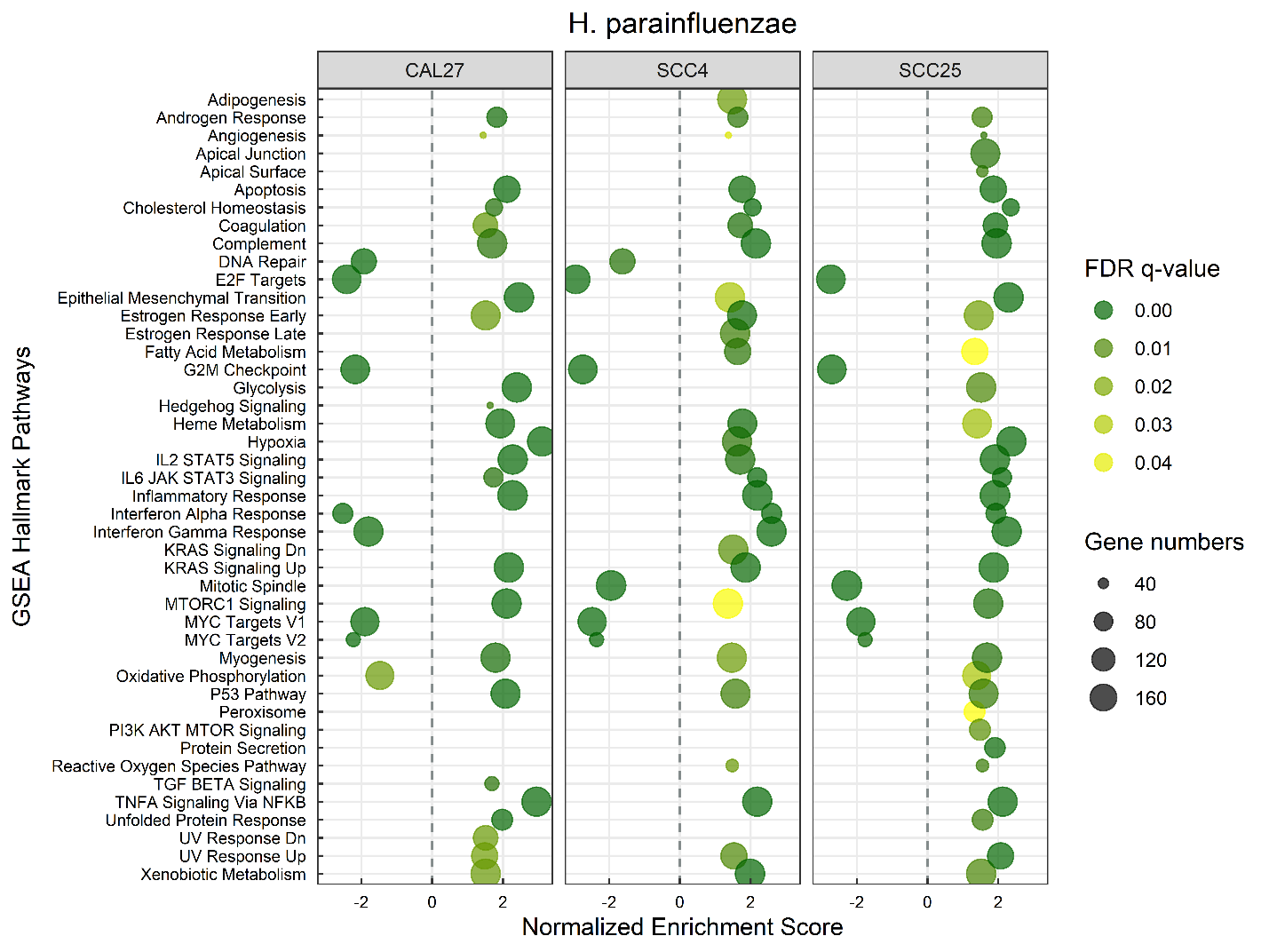


**Supplementary Figure 6.**  **GSEA Hallmark Pathways significantly altered by *Haemophilus parainfluenzae***. Microarray data obtained from co-cultures of OSCC cell lines with S. mitis were analyzed with Transcriptome Analysis Console to identify differentially expressed genes (DEGs), which were in turn processed with Gene Set Enrichment Analysis (GSEA) to identify significantly upregulated and downregulated pathways (FDR ≤ 0.05).


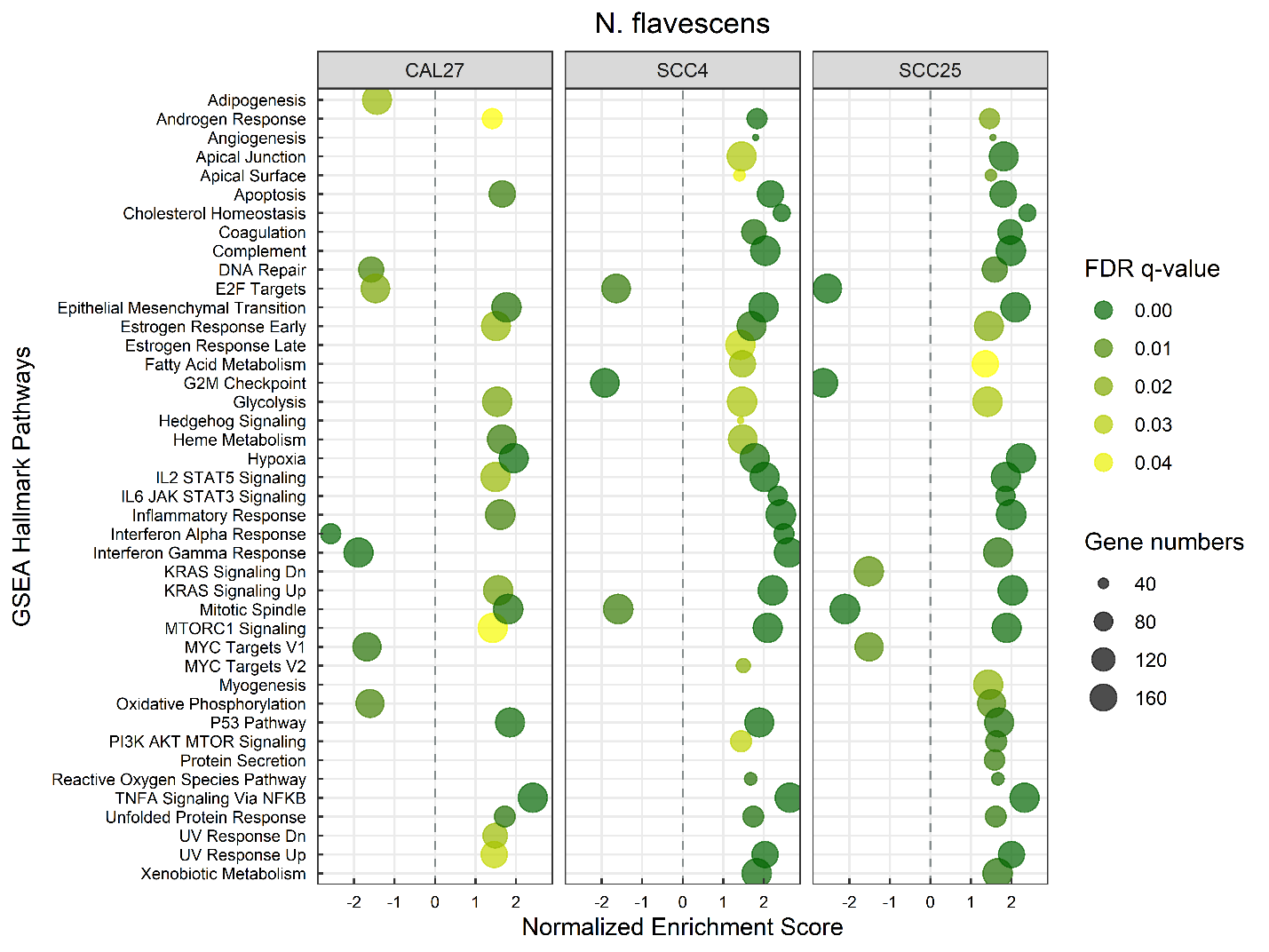


**Supplementary Figure 7.**  **GSEA Hallmark Pathways significantly altered by *Neisseria flavescens***. Microarray data obtained from co-cultures of OSCC cell lines with S. mitis were analyzed with Transcriptome Analysis Console to identify differentially expressed genes (DEGs), which were in turn processed with Gene Set Enrichment Analysis (GSEA) to identify significantly upregulated and downregulated pathways (FDR ≤ 0.05).


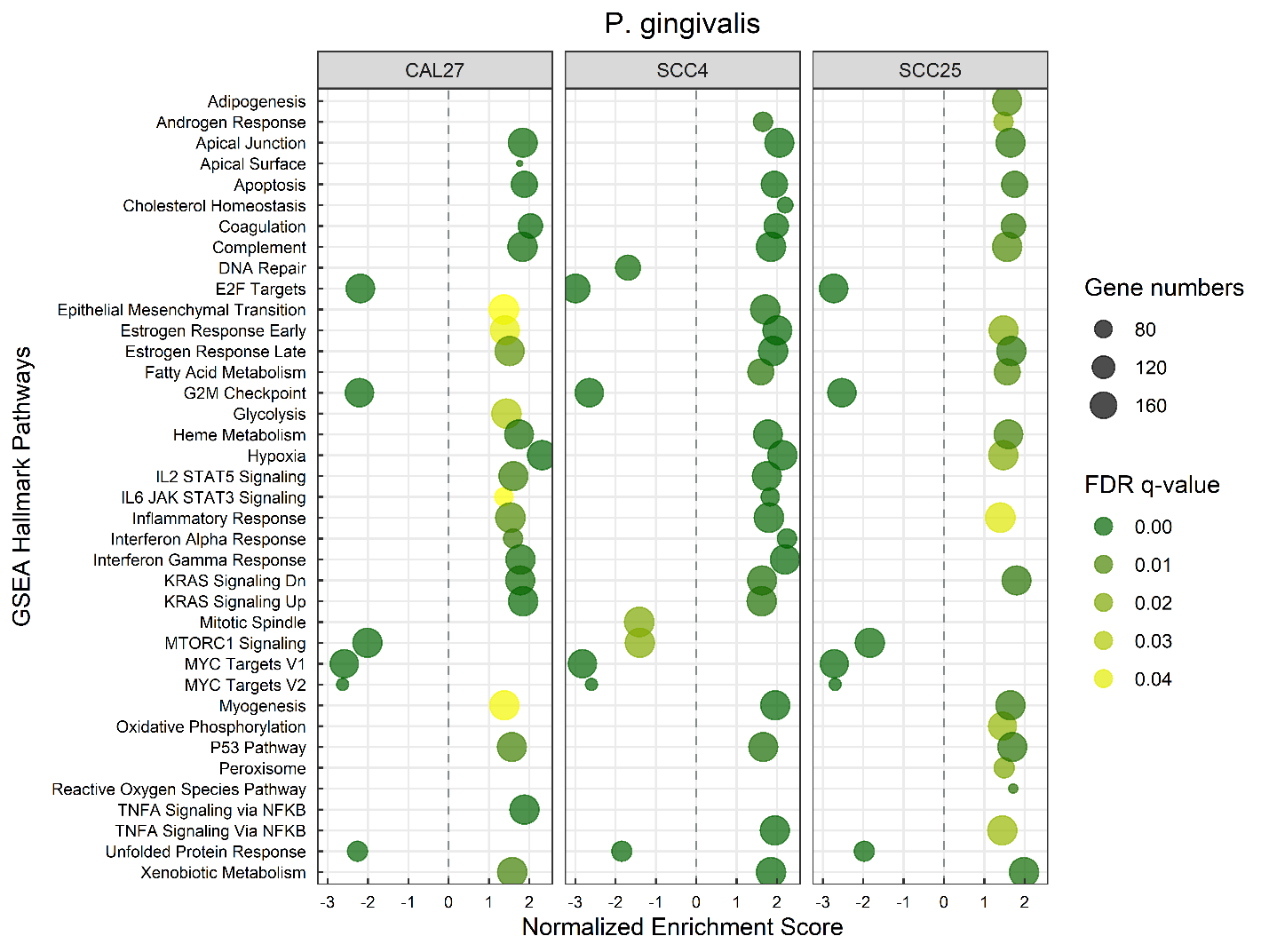


**Supplementary Figure 7.**  **GSEA Hallmark Pathways significantly altered by *Porphyromonas gingivalis***. Microarray data obtained from co-cultures of OSCC cell lines with S. mitis were analyzed with Transcriptome Analysis Console to identify differentially expressed genes (DEGs), which were in turn processed with Gene Set Enrichment Analysis (GSEA) to identify significantly upregulated and downregulated pathways (FDR ≤ 0.05).
